# Valedictory Delivered before the Graduating Class of the Baltimore College of Dental Surgery, March 1, 1855

**Published:** 1855-04

**Authors:** Wm. H. Dwinelle


### ARTICLE IV.

*Valedictory Delivered before the Graduating Class of the Baltimore College of Dental Surgery, March* 1, 1855. By Wm. H. Dwinelle, M. D., D. D. S.

Gentlemen of the Graduating Class:

Although I realize the importance of the office I have assumed in addressing you, and know how far I shall fall short of expressing to you all that I desire to say for your good, still, it is an enviable pleasure that I now feel. For a moment I seem to sustain here a dual relation. I look upon your young, earnest and hopeful faces, and I am carried back to the years that are gone, when I set out upon the great experiment of professional life. Gentlemen, I sympathize with you, I feel as you feel, I know your anxieties, your hopes, your resolves and your wishes. I know your earnest desire to learn the best way of attaining to the largest sphere of usefulness, and standing eminent among men; I know the eagerness of the youthful heart, and the enthusiasm of the untried spirit to press forward to the realization of its hopes of glory. I know the anxieties and expectations of this hour—this *last* hour—to gain from the lips of the speaker some crowning words of advice, which shall be as a grand recapitulation of all your instructions and your labors—burning words, wrought out from the severe experience of those who have preceded you—words that like a herald shall seemingly come back to you from *your* future, ere you start on your mission. Words, that, lifting up the half drooping energies of the spirit, or fortifying the strong hopes of the hour, shall hereafter come back to you in the still watches of your patient and lonely toil, and whisper peace and hope and courage.

I participate with you in all of these feelings, and with you I am looking up from your seats to know what the speaker shall say! What shall the speaker say, and how shall he best say it! Gentlemen, it is arrogance to assume that I am equal to this, and yet, such as I bring, it shall be my pleasure to lay before you. For I hold that the highest pleasures are those which spring from the opportunity of giving advice and instruction to those who are to follow in our footsteps, to work out the same burning deeds and thoughts, and to struggle with the same destiny which has brought tears to *our* eyes, pain to *our* flesh, and the *test* to *our* sinews. It will be my purpose to warn you, so far as I am able, which paths to shun, where shoals and quicksands lie, to point you to the signal towers of experience, and to illumine with my feeble lamp the pathway that lies before you.

You now stand at the threshhold that leads out into the broad and untried world. You have accomplished one grand effort of your life, one to which you have patiently, earnestly and hopefully looked forward through years of toil and privation; for a while you repose upon your first laurels, and rest by the way side; then to gather up your armor, and, with manful hearts, to go forth into the great battle of life; a life which must be made up of a continual succession of efforts and of victories; for no sooner will one adversary have been overcome than another will present himself to wrestle with the energies of your being—in turn to be subdued and give place to another; and so you successively strive on, become victorious and step forward again to grapple with new foes, which shall as continually take the place of others, to the end.

I know that this is a gloomy picture to present to the youthful mind, to your warm and hopeful hearts. Nor would I for a moment dampen the ardor of your faith, but that I may call out the true and healthful energies of your nature, to meet with their largest advantage the inevitable destinies of life, for "life is *real*, life is *earnest*," there is no royal road to true success—besides it is a *glorious* warfare, it is a continual combat for *truth*, and yields its own sweet rewards. Indeed, the most substantial joys are those which flow from the consciousness of striving for the supremacy of truth, no matter at what cost. You realize that your efforts are heaven-approved, for they are linked with the mission of the Divine.

Do you inquire what *is* truth, and how the sentiments just uttered can be applicable to your case? Truth is so broad, so high, so deep, has so wide a range, that it underlies and runs through—*every thing*. It is a grand principle of heaven, which, pervading all the laws and principles of the universe, is continually struggling for its own recognition. Truth is light contending with darkness, whether in the moral or physical world. Truth is wisdom struggling with error; virtue with vice; knowledge with ignorance; honesty with corruption; the right way with the wrong way; beauty with deformity; integrity and skill with rudeness and quackery.

It is your duty to develop the highest truth in your art, to separate it from the errors and confusions which surround it, and present it to the world in all its practical beauty. It is for this that you entered these halls, for this that all practical arts are here enlisted and made accessory to your education; it is for this that you have labored early and late, hand in hand with your beloved teachers, and it is for this, that you now garner up all of your advantages, and go out into the broad and inviting fields of future usefulness.

There is nothing more beautiful than truth, nothing that is so universally applicable to all of the phases of life. There is no art, no science, no law, perfection, harmony, order, beauty, no creation, no true success, but is built upon truth, but leans upon it for support, and recognizes that all its exaltation and integrity is by virtue of this grand and immortal principle.

Did it ever occur to you that every new fact, every new creation, was a new truth; every new discovery was a new truth—a glorious truth, harmonizing with and becoming a part of the great *oneness* of all *truth?* What a high destiny is his who

17*

enters most largely upon this great mission! Every new acquisition to the grand accumulated store of *truth*, reflectively expands and elevates the mind of the discoverer, and swells the fountain of which all may partake.

Make the application to yourselves and consider how fortunate it is that you have engaged in a comparatively new field which promises such great developments.

Gentlemen, I congratulate you upon having completed your preparatory course of education, and I welcome you to the brotherhood of the profession—a profession, though eminently practical, still, a profession of students and learners like yourselves. Do not think that you have finished your education. Believe me, you have just begun, just taken the initiation, just learned how to educate yourselves.

We welcome you to our fraternity. We have need of you, for you come to us possessed of large and unexampled advantages, which no one of us could have commanded; which no wealth nor position, nor favoritism could have secured, you are the happy recipients of the entire harvest of all the years of experience which our profession has known from its first dawning to this hour. A profession whose history, though unparalleled in its results, is still more remarkable for being almost embraced within the memory of some of those who now sit before you. Above all others who have preceded you, you have cause for congratulation here to-night. You receive your commission from the first Dental College in the world—the first in every sense, the time honored pioneer in an enterprize so dear to our interests, and which is now shedding such lustre upon our art. You have received the advantages of another year's accumulated store of experience; a year abundantly fruitful in its harvests, and you form a class exceeding in numbers by far any that ever graduated before, of its kind.

You are chartered by this institution to go forth into the world, with its full honors upon you, to enter the broad and still widening fields of labor in which you have chosen to act, you are enjoined to exert your best energies and your skill to alleviate human suffering, to arrest the processes of time, and stay the encroaching and subverting elements. What a high commission and what a noble work lies before you!

The advantages of position were never more fully realized than in this first hour of your professional career; as you pass out into the world from your beloved *Alma Mater*, graced with her honors, gifted with her teachings, and richly laden from the free storehouse of her experience, remember, that out of, and above all of these, springs a higher and a still nobler gift; paradoxical as it may seem, she gives you *time!* Her superior facilities enables her in a large sense to invert the natural order of things. She carries you forward through the plodding years of those who have grown gray in their toil, and gently places you down co-workers among them with your youth, your hopes and your energies entire. You find yourselves suddenly, as if by transition, occupying a vantage ground whose theory embraces all that any can command, and whose practice in many respects is only excelled by the largest experience. The experience of age is combined with your young manhood, and nothing is wanting but the exercise of your fresh energies, to develop high results in our art hitherto unattained. There are many hidden mysteries in reserve for us yet; unwilling nature shall yield still again to the persistence of the earnest votaries of our science, and crown their labors with "many a gem of purest ray."

It is for all of these reasons that we expect much of you, our art is young and you are young; soon you are to take our places. This day you assume a charge and a responsibility of a most momentous character, the interests of the profession, its exaltation and its success is in a large measure, committed to your hands. Guard well the trust!

I have congratulated you upon your being graduates of this College; in so doing, I would not disparage other institutions of its kind. I honor them all, for as the interests of our profession are inseparable, so the honor and success of each of these, must ever shed additional glory upon an institution from which they are all pleased to acknowledge their origin.

Our Dental Colleges are no longer an *experiment*, they have become a necessity, and are almost universally recognized as literally one of the *institutions* of our profession. The wildest hopes of their original founders have become more than realized. Their influence here can be somewhat estimated, but their influence upon the profession, and upon our co-laborers abroad is past computation.

One or two efforts were made to establish a College, like our own, in London, and although large and praiseworthy influences favored the enterprize, yet it fell to the ground without leaving a hope of the subject being revived for years to come.

As one of the evidences of the estimate in which the Degree you to-day receive is held abroad, I may state to you, that Mr. Thomas Arnold Rogers, of London, a young man of high moral and professional excellencies, one who is abundantly successful in his profession, told me some two summers ago, that were it possible for him to leave his practice at home, he would willingly cross the ocean and labor within these walls for years, if he could return graced with the well earned honors of the Baltimore College of Dental Surgery.

I have said that dental colleges have become a necessary institution of our profession. Strike them out of existence and you place our art back far behind the years of its most successful progress—worse still, you doom it to a singleness, a feebleness of purpose and undeveloping apathy, a blind and jealous conservatism, from which no time could redeem it.

The fable of the bundle of rods, and the moral of the strength of union, is familiar to you all, and it is true as ever. The advantages of association and combined labor, are too apparent to need more than a bare reference to them. There is a magnetism of contact, full of creative energy. Flint and steel are passive of themselves, but clash them together, and warmth and brightness dazzles upon the sight. "By means of difference and discussion, and by interchange of thought and opinion, life and vigor is elicited, and the conversation sparkles. Style gets a fascination from antithesis, zinc and copper taste sweet together in the mouth and give forth a flash." The positive and negative poles of a battery never come together without producing a

*result.* By friction of different mental organizations together, an idea is evolved, a new law is discovered, a new creation is added to the wealth of knowledge, and the long coming rays of a new truth, like those of a far off star in the laboratory of heaven, reaches and illumines the world. You have done an hundred things together this winter, that each and all of you would never have done alone.

The influence of association, reaches, too, into your moral and physical natures. It teaches you of your common humanity. Mountains and seas divided you, you were strangers, and the spirit of narrow jealousy, unfaith in man, isolation and distrust dwelt in your bosom. You come together, and found for a *surety* that "God had made of one blood all nations of men to dwell together on the face of earth." You now feel that you indeed belong to one great brotherhood. You are friends, for you recognize in each of your natures, your own reflected heart; and love, and faith, kindred feeling, and wide reaching charity sit enthroned in the temple of the soul. The spirit of man hath growth under these influences, and aspires higher toward heaven. I believe that you individually feel that you are better morally, as well as mentally, for having come together here. Cherish these refining and beautiful fruits of union, nor let them cease to dwell in your natures while you live. Why should you not? though your personal intercourse is broken up, perhaps forever here—why should you not keep the union of spirit here established entire to the end. The soul is not of time or of locality. The sweet bonds of union, the golden chains that link the interests of your faithful and beloved teachers with your own—let them never be broken. Ever keep alive dear remembrances here formed, until you meet face to face in that great sanctuary of God, where the heart realizes all of its affections and aspirations, and where the expanded soul dwells with the Infinite.

Successful union naturally presupposes direction to a specialty, or thing to be accomplished. A union which encounters endless diversity, finds itself outnumbered and succeeds, in nothing. This proposition when taken in connection with the large field of our specialty, is sufficient to prove the *necessity* of Dental Colleges to our profession, as well as their superiority for giving the requisite instruction in our art. It is folly in some of the advocates for a Dental Chair in Medical Colleges, to assume, that thereby, any thing more than a very general light could be thrown upon our art. As well might we profess to teach the *whole science* of medicine in that collateral department of our course—such a thing were impossible and we should fail in both.

Gentlemen, this is not an exhaustive essay; time and professional avocations force me to give you only glimpses of a few portions of the wide field before you. A satisfactory examination of its diverse and ever expanding surface, is the labor of a life; these glimpses will, however, point out many glorious vistas which may be pursued at leisure.

Other considerations now invite our attention. If your professional hopes swell upward towards an exalted consummation, I must remind you that no degree of skill, ingenuity and industry in your profession, will atone for the large class of qualifications to success, which partake of a personal character. Few callings bring their votaries so directly into close intimate contact with their employers, as does ours. It is for this reason that no man, equally with the Dentist, can derive advantage from the symmetrical combination of manly address, propriety in attire, intelligence and high courtesy which goes so far to constitute the gentleman. As from no treatise can you learn specific rules for guidance in all emergencies, so there is no school or art for making true gentlemen. Such a character is not made; it *becomes*. It is not a process, but a result. While urging you to desire a pure mind and personal neatness; to preserve the freshness of your youthful feelings through life; to cultivate a love of the good, the true and the beautiful; to nourish your contempt of wrong and low-bred pride; to be impatient of tyranny and all domination of vice, though gilded and set in high places; to strengthen, by due exercise, those generous emotions which every right thinking man delights to honor; and, above all, to dare to be yourself on all occasions, I am advising you to achievements impossible for unaided human strength. From the vantage ground of christianity, the paths of duty, honor and temporal advancement, which are thought to be diverse when viewed from below, are perceived to unite and thence forward become indissoluble. Only in that elevated path are found peace, content and usefulness.

About to depart from these halls, with the deserved honors of this Dental College, and assume the varied and arduous duties of professional life and citizenship, you are inexcusable, if you have not maturely scanned the momentous questions of human destiny. No qualities of the head, or cunning of the hand, or endowments of person, can absolve you. The fleeting days of pupilage will end with this discourse, and thenceforth you will be clothed with the rights and privileges of men. Each word and act will then, at a bound, have acquired a new weight. No weak or low scheme of religious faith can guide you safely there.

Wherever placed you will not lack instruction in your great religious duties. But where can you learn the nature and worth of those minor morals, called manners? No definition can describe them, no toil can secure them; they spring from the man and the occasion; they are the offspring of a kind, genial nature, informed and directed by a ready tact, which imparts to them a flexibility adapted to the moment and the object in view. Your habits of personal neatness, your taste in office fixtures and adornments; your behavior in the drawing-room, and your carriage among men, are valuable auxiliaries to professional success. A neglect of these matters marks the sloven and is a reproach to our profession. I cannot dwell upon the disgust excited by such habits. I need not enlarge upon this. He has mistaken his calling, and the want of employment will coerce him to seek other means of livelihood. It is not trivial, then, to give thought to these minor morals. Our patients are from all classes: and a taste shocked or a fancy gratified, is often business lost or practice won.

Nor, will you, if wise, neglect to acquire large acquaintance with many subjects, arts and sciences. As aids to your success, you should become deserving of the friendship and esteem of your patients, and to this end you need the ability to become men of weight in society. You cannot fail to perceive how much it will add to your usefulness, to cherish such arts and habits as will heighten your self-respect, and render you an equal, and a valued acquaintance of the learned, the virtuous, and the refined. Acquirements of this nature may, with reason, be prized less as pledges of business, than as honorable guarantees of usefulness as men, and as an earnest of high position and renown.

I advise a generous culture of literature, art, and pure science, if practicable, not more for themselves than for their tendency to redeem your manners, language and habits from the pedantry of the college and the office. Think how the language of the clergy often betrays, in its phrases and illustrations, the peculiarities of the ministry; how the lawyer often disfigures his speech by technical terms, and how the *mere* physician has his vocabulary of professional words, which cast a pedantic hue over his conversation; how the tailor sees in Niagara Falls only a "nice place to sponge a coat," and another person a "capital spot for a saw-mill;" how, in short, the members of all trades, professions and pursuits are more or less liable to be infected with the dialect of their business and to permit their thoughts to wear circular channels for themselves. Reflect how this corruption of speech impairs the purity of our Saxon tongue; consider how this vicious practice of viewing the glory of art; the beauty and grandeur of nature; all pleasures of sense and intellectual joys, through a professional medium, indicates a descent from the standard of gentlemen to the obscure level of a mere class. To this vitiating influence you have been exposed, and unless it has been counteracted by a parallel and independent course of study, and by habits of original thinking, by the liberalizing tendencies of the fine arts, and the delights of society, your pedantic forms of speech and professional air, will supersede other advertisements of your vocation. The system of self-culture which I recommend, exacts that you become accomplished dentists, and also, makes you educated men of the world, with no marks of segregation from the common pursuits and ordinary habits of educated men everywhere.

Not for an instant are you authorized to imagine, that the professional utility of good morals, correct manners, and a broad, comprehensive education in various departments, not immediately related to our calling, has been adverted to from a covert opinion that our profession is not one of which we should be justly proud; nor from a belief, that in order to be respectable, the dental art needs the reflected light of other and meaner—perchance nobler—arts or sciences. I acknowledge nothing of this. If masters of your profession, you should be as proud of it as the lawyer, the sculptor or warrior of his. Not for its age is your profession eminent, and its devotees placed high among the benefactors of the race. As an art, still more as a science, has dentistry made giant strides during the last quarter of a century. The great explorers of the dental world, are yet gracefully leading lives of affluence and honor; some of them kindly cheer us with their presence to-night. They have not exhausted this great field; there remains much to learn and to explore. No dentist, however distinguished, can long retain his superiority in our ranks, unless he continues to enlarge the domains of his art. No neophyte can rise to high station, if he sink down into inglorious sloth, content with the study of what others have written and done for his instruction. There is yet much to be accomplished; desiderata there are, enough for the exercise of industry, skill, patience and invention; enough to bring within the bounds of possibility the realization of the wildest hopes.

The laboratory lays hold on chemistry, on painting and sculpture; the operating chair is allied to the surgeon's and the physician's art. How these profound sciences and sublime arts are tributary to our profession, and of their transcendent value to it in its every department, I will presently speak more in detail. I allude to these things here, that you may perceive how our profession bases all its claims to culture, public respect and esteem, on its solid attainments in useful science, and its constant use of the noblest principles pre-eminent in the fine arts.

Hitherto, education, taken in its widest and loftiest sense, has been considered as an integral element of professional success.

The argument for thorough education was, for the moment, rested on the low ground of pecuniary gain, in order that, at the proper time, I might, with the more force, impress you with a juster sense of its reflex influence on yourselves. It would be a wrong to you and to the subject, not to view it from a nobler stand-point.

We all need to more than live; to do something nobler than to grow weary and starve every emotion and capacity down to the routine of task-work. To grow old in the laboratory and over the operating chair, with a sordid motive; if this is your idea of life, better that you had taken to breaking stones on the highway. Getting food and raiment is not the sole end of our profession. Is there no compensation in the fact that it ministers to health, beauty and utility? Is there no pleasure in the gratitude of patients; in the gratification of artistic tastes, and in the necessity which our art imposes, of a steady improvement in directions that refine the heart and enlarge the understanding? A little of thought, and a slender stock of knowledge will enable you to vegetate, and perhaps to get rich and become not hated—for we do not hate such *androids*—but despised. You have not been pressed to covet education simply as a help to business, in order to swell your income and enlarge your list of patients. And yet it is desirable for what it does in this direction, because through its aid, your support being secured in less time and with fewer hours of labor, you will have the greater leisure to develop your intellectual and spiritual natures.

It is the glory of our profession that it yields opportunities for high and refining self-culture, helping to make each sense a perpetual joy, and each faculty of mind a song of swelling gladness. To all, education is what sculpture is to the block of marble; which with equal ease produces a Silenus or a head of Jove, an Apollo, or a Hecuba.

From irregular native capacities, passions and instincts, education will develop a mind of symmetrical proportions; it will bestow strength, grace and beauty; supplying aliment for healthy growth, and elaborating and diciplining each faculty.

To this true and inner growth—this steadfast process in training each energy, I counsel you to bend all your efforts, until your tastes are in harmony with whatever is true, or beautiful, or good; and your whole moral and intellectual nature cordially responds to whatever pertains to our common humanity. Toil under the sharp whip of necessity, gives rugged strength, self-reliance, boldness, and the ability to do and dare great deeds; whereby life becomes an epic in action, grander, sweeter, of more mournful pathos, of more purifying influences, than anything yet sung by old or modern bard, in hall or bower. But leisure, travel, observation, study, plain living and high thinking, confer tasteful accomplishments, a quick eye for the beautiful of nature and of art! They contribute wit and song, and hearty potations to the garlanded feast of life. You know by what small means a man may be changed fundamentally and forever: the memory of a mother's love, a glorious line from some old poet, dead a thousand years—the peal of solemn bells—the death of a friend—a gleam of rarer beauty in lake or sky, or a suddenly remembered strain of gladness.

But where, gentlemen, in the long round of professions, of arts, of sciences, can be found one which is surrounded with worthier or more abundant influences, and which may contribute more toward the formation of the man for all time, than does ours? We should be co-workers "with poets, historians and statesmen; with the lovers of the true, the just, and the useful; with the searchers into the mysteries of unwilling nature, making the winds and the waters, the palpable and impalpable essences of things, tributary to man; creating new gratifications for the body, and developing new capacities in the soul."

Gentlemen, the transcendent weight and importance of the considerations which I am discussing, impel me in passing to treat some of them more in detail, and perhaps to clothe them with a more practical character.

Cultivate the arts as well as the sciences. You not only can make them indirectly applicable to your profession, but most directly so to yourselves, contributing in an eminent degree towards building up and expanding the capacities of your being. Devote occasional leisure hours to music, poetry, painting and sculpture: their influence is ever elevating and profitable. There are silent heaven-born emotions, dwelling in the depths of every human soul—emotions, too deep and too delicate to bear the chains of ordinary language; inspirations of the divine, the beautiful and the true, which, whenever they do struggle up through the grossness of our mortality and find a voice, exhale heavenward, and bring back to us, like an echo, a benison to refine, to exalt, and to assure us of the immortality that lies in our future. These emotions are ever striving for utterance, wrestling with our natures for language, and appealing for expression. Where human language fails, the higher, the diviner arts, with their inarticulate but eloquent voice, come to our aid. Music, with her melody, voices the immortal longings of the soul. Painting, with her ideal beauty and harmony of color, pictures the angel visitors and golden-tinted visions of our dreams. And sculpture, with her silent forms, graceful mould and intelligent features, bathed in a holy light of her own creation, speaks in a language more full and eloquent than words, *satisfying* the yearning spirit with her inaudible responses, and ministering to the needs of the soul like emanations from the divine.

These are the voices, which, exalting and purifying our inner natures, build up the God-like within us, and point us heavenward, where *new* senses shall be given us, and where we shall revel in the fulness of complete expression.

The influence, the teachings, and the ministerings of the beautiful cannot be estimated. The beautiful and the true are the grand themes of nature, and should find expression in all her instrumentalities; all her voices, all these harps of a thousand strings, should be attuned only to the beautiful and good. It is the continual effort of all art to personify the beautiful, representing the human, reaching upward toward the Divine, and struggling to express those immortal longings which lie deepest in the soul, expanding it to a wider sphere of action, and a more enlarged comprehension of a still higher, more beautiful and more true. The beautiful, embodied in the works of art, in harmony, in order, in neatness, in dress, in action, in thought and conversation, is an exponent of the mind of the individual who worships at its shrine. Here it realizes its true mission, its impress on the soul.

Adorn your office with works of art, even though they be cheap ones. If selected with good taste, a trifling expense will go far towards beautifying and rendering cheerful the place where the most useful hours of your life must be spent. We do not know how much our actions, our happiness, and our character are influenced by the very rooms in which we live. We owe it to our patients, also, to use every adornment, and enlist every pleasant device to compensate for, and allure the mind from, the stern necessities of our profession.

It is one of the legitimate duties of our art, to study the beautiful in form, expression, harmony, color and effect, symmetry, and keeping, the balance and relation of combinations, especially in the mechanical and corrective departments. No clay in the hands of the artist was ever more subject to his control, than are the varying expressions of the mouth and its surroundings, subject to the controling influences of our art, which become, in a large sense, akin to that of sculpture.

The mouth, the most interesting and important of all the features, where intelligence, in silent expression, sits enthroned; where character leaves its impress, and where the heart and its affections find a voiceless utterance. In supplying a complete denture, you can make the mouth agreeable, or forbidding; restore the lost individual expression, the lost beauty and inviting grace, or make it incongruous, inharmonious, and repulsive. If we were responsible for the discordant and unhappy natural expression, which disfigures the countenances of those we occasionally meet, how fearful it would be; and yet there are many in our profession, who, through ignorance or want of artistic feeling, or a proper appreciation of the above elements, have gone deliberately to work and literally constructed just such artificial deformities, causing a man to misrepresent his own character and disposition, and placing in his mouth a continual libel on himself. But in the corrective and restorative department of our art, that branch which has for its design the correction of malformation and deformities, a still higher field is reached. Beauty and symmetry is the rule; deformity, the exception. Congenital malformations are in danger of transmission, but if corrected or restored by art, the future unhappy tendency is almost sure to be destroyed. What enviable opportunities are yours to arrest and subdue an afflictive and repulsive conformation, and relieve the future from its curse.

'Tis yours to beautify
And yours to bless. T' assuage the stinging throe
Of pain; bid suffering cease, and pour the oil
Of comfort into many wounds. The lost
To restore, deformity to raise up from
Her humble path obscure, that she, with smiles,
May take her unaccustomed place among
The sons of men. I charge ye, that to you
The noble boon is given, to model o'er
The human face divine, on which
The signet seal of Heaven hath fixed her impress true.
Be cautious then, nor e'er with careless and
Indifferent hand, abuse thy honored trust.

A casual consideration of the innumerable reflex relations which the legitimate practice of our art has upon the whole system, throughout all its ramifications, tends to impress upon us the vast responsibility, the almost sacred trust which lies in our hands. We are to make impressions for good or ill, mental as well as physical, whose influences shall reach forward into the undeveloped future, and whose vibrations shall be harmonious or discordant to the end of time.

It seems to me that here is a fitting place to speak of what may be termed our Professional Code of Honor. It is a theme which expands before my mind to an extent commensurate with its great importance, and I feel impatient to dwell long and emphatically upon it. It might well be the subject of an extended essay; but time forbids that I should say more than a few suggestive words to you, and pass on.

The relations between ourselves and our patients must always be calculated to keep in exercise the most exalted and honorable feelings of our nature. Under many circumstances a high degree of confidence, and an almost sacred trust is placed in our keeping—a trust, which, while it appeals to our honor as men, is highly complimentary in its character, and is calculated to heighten our respect and deepen our esteem for the subject of our charge. Humanity sometimes comes to us in its worst phases—indeed, the gentlest natures, when shattered by incessant pain, enfeebled by disease, or prostrated by long vigils with suffering, often misrepresent themselves and permit the spasmodic and uncontrollable throes of a rebellious and pain-goaded organization, to be the exponent of what they are not. A hundred similar cases cluster before the mind of us all at the bare reference to the subject—all of which appeal to us as gentlemen representing an honorable profession.

The man who would so far violate his own honor and degrade his calling, as to abuse the confidence reposed in him by his patients, even in the least, is mean and perfidious, and deserves to be frowned upon and cast out from association with all virtuous and honorable men. He merits not success; his commission should be taken from him; he should be drummed out of our ranks and become compelled to gain an humble livelihood among felons and outcasts.

Among lawyers, this high code of honor is regarded as most sacred, and should any member of that profession violate the trust of his client, he is *broken*, thrown over the bar, and degraded. So exalted an estimate is placed upon this manly principle, and so essential is it considered to professional character, that, at some of our first Medical Colleges—and I honor them for it—a student is not permitted to graduate, no matter how excellent his scholarship, or how skillful his hand, until he makes oath that he will never, under any circumstances, betray the confidence of his patient. Be this a solemn hour in which you shall take the same pledge to yourselves, and I charge you, keep it as sacred as they.

In this connection, I must not fail to remind you of the courtesies due to our patients; those kindly, considerate courtesies, adapting themselves to the varying phases in which afflicted humanity presents itself for our aid. Their exercise is especially called out in ministering to the misfortunes of our fair friends. If you have a mother, sister, wife, or the memory of these dwells in your hearts, take the lesson home to yourselves, and consider that the trembling, sleepless, pain-worn woman is somebody's wife, sister, mother; be gentle with them as though they were your own, and thank God that you have an opportunity of alleviating one woe of those to whom a mysterious Providence has given so large a share.

Never deceive a child, nor stoop to the low trickery based upon a lie, which is so often the resort of the unthinking and unprincipled, to outrage confiding childhood, and surprise it with a grievous pain of heart, with the lesser one which crashes upon its delicate head from your rude and hasty hand. Once gain the confidence of a child, and if you choose, you can keep it always—once lose it, and you cannot regain it. You make an impression that remains forever. A few to-morrows hence and the child has grown to manhood, or to womanhood, and then you will find men and women making an estimate of your character upon a cruel lie, which has ever darkened their first recollection of you. It is not necessary; the heart of trusting and loving childhood is full of chivalry and noble pride, if you but use the right means to reach and develop it. Lay the whole truth before them, and tell them to be a man and endure it as father does; be a woman, a lady, like mother, and bear the momentary pain. You have touched the right chord; the smiling face, and the glance of the large and lustrous eyes upon you, bespeak it, and affirm their young energies are all braced for the contest; and they go away with a larger love for you; there is a bond of union, of good faith between you, a confidence and respect, which shall warm towards you when you have grown old.

The influence of these petty deceits do not stop with the child; they injure you; for every act like these, trifling though they may seem, cast another shadow upon your moral nature, gradually undermine your integrity, until, before you are aware, you find yourselves clothed in the garments of deceit.

Permit me to diverge here for a moment. Our profession heretofore has been degraded, and is still in danger of being often misunderstood. Our intimate relations with our patients afford an excellent opportunity to correct many erroneous impressions, to give them just views of the multifold relations of our art, and educate them to a practical appreciation of the magnitude of its character, to be transferred to those with whom they will be associated, when they go out into the world. There are many popular errors among the masses, and there is much inexcusable "Ignorance of the learned" in regard to our profession, which ought to be corrected. Without being pedantic or obtrusive, you can impart much practical information to your patients, information of an intensely interesting character, which will come home to them as it does to ourselves; while, at the same time, it will go far towards overcoming the evils to which I have alluded, not only directly, for the moment, but in instituting an inquiry and investigation, on their part, which shall result in a material aid to our onward progress.

A popular writer, not long since, gave a glowing account of one of her friends who had a lost front tooth restored to her entire, by chewing charcoal for a few days! A clergyman once desired me to remove the *salivary tartarus* from his teeth! Medical journals have published learned articles about men, whose entire dental arch was made up of molars! There are a thousand errors prevalent, and a host of misapplied and unmeaning expressions daily falling from the lips of those who frequent our office, which we should correct. Our own vocabulary, too, needs renovating, and, without being over-technical, we should be sure to call things by their right names. Above all things, avoid *slang*, in all its forms. If it is not always indicative of low origin, it is ever a sure index of a vulgar and slovenly turn of mind, which strives to be facetious at the expense of our noble language, by wasting it upon a stupid combination of silly words. A man is known by the insignia of his daily life, his conversation, and his actions. Put on no such livery as this.

To return. Let your intercourse with members of your profession be characterized by the widest spirit of liberality and manliness; keep no secrets locked up within a selfish bosom.

Remember that you owe a debt to the past and to those who have preceded you, which you can never repay. You hold in *trust* what has been freely given you, and you are responsible for its right application. Besides, all that you are, or can be, the use of your highest faculties, your most elevated conceptions, your talents, best efforts, productions, energies, belong to the world. In giving them to the profession and to mankind, while you pay a part of this natural debt, you reflectively benefit yourself.

Towards your enemies—and the best have them—there is a courtesy due; the courtesy of charity and of silence. Do not be disheartened at their unhappy importunities. If you are in the right, they certainly are in the wrong, and for them to hate you is to pay you the highest compliment; it is a virtual endorsement of your position. Their nature is discordant and jars against truth; they hate you by virtue of their own oppugnancy to your goodness. Let your highest revenge upon them be to "let them alone, most severely!" Always have a high moral character—tempered with a christian spirit—to fall back upon, and you are secure through all trials. Stand by the divinity of your nature, and remember that no one can do you any permanent harm but yourselves; for what is envy but bitterness that drinks itself; hate, but the morbid beatings of an aching heart; slander, but a harmless viper that writhes under its own sting; insult itself, but a poor weak breath upon the wave of time, from pallid lips that die ere the morrow. None of these can ever hurt you. The annals of eternity will show that all of the sin-marks and soul-stains, that have survived the blanchings of time, are your own.

Never do wrong with the expectation that it will never injure you, if your indiscretions are not known of men. Retribution is sure to follow, it needs not superstition or belief in special providences to assure us of this; if not in striking manifestations, yet in the blunted moral sensibilities, in the loss of the higher pleasures of virtue, and in the fearful retrogadation downwards and assimilation with brutes. God has made it so from the beginning. The subject of retribution becomes the abandoned slave to his own vices, and having nothing but himself to lean upon, derives his only enjoyments from a changing, barren and deceitful world. The penalties of the moral law, are as immutable as are those of the physical; the harmony of justice and truth renders it inevitable.

When the material and sensual triumphs over the ideal and intellectual, when the high moral senses are benumbed and cease to lay hold on the heart, when the divinity that is within us, and charity, and goodness, and love, and all the now weeping train of virtues, are banished and crushed out of our natures, then manhood dies, then the gifted soul is laid down in the dust, and nothing but the animal remains.

A distinguished orator once said, "Justice is a theorem; punishment is as exact as Euclid; crime has its angles of incidence and its angles of reflection; and we men tremble when we perceive in the obscurity of human destiny the lines and figures of that enormous geometry which the crowd call chance, and the thinking man calls law."

The literature of our profession is yet to be formed; it must be mostly committed into your hands and those who are to follow you, to develop, and while earnestly inviting you to enter upon its high pursuit, I would warn you against engaging in writings of a controversial character. They are generally unprofitable and not worth the paper they are written upon, nor the time wasted in their perual. In building up our literature you have a noble inheritance as a basis to work upon.

I caution you against listening to the weak whinings of those who imagine our profession is not equal in dignity with others. A profession which is honored by such men as Hudson, Parmly, Harris, Maynard, Townsend, Arthur, Dunning and others, can never be an humble one. Whatever be your occupation, it is dignified according to the amount of honesty, skill and industry you bring to it.

It becomes you to dignify the profession, your Degree will avail you nothing unless your future life shall prove that you are equal to it—you should do more than this, you should strive to be superior to it, dignify and exalt it, so that when future aspirants shall knock at the door through which you have been permitted to pass, they shall realize that it is no mean ordeal that awaits them, before their names may be recorded with the high and honorable ones which have preceded them.

Quacks are so proverbially in the wrong, that it is almost a safe rule *not to do any thing which they do!* Do not warrant your work. Do not publish a flaming advertisement, filled with ridiculous testimonials from ex-justices, decayed greatness and ignorant nabobs. Do not solicit patronage. Do not *be* patronized. To warrant your work is to warrant care and cleanliness on the part of your patient; to encourage their slovenliness and ensure them against disease and the effect of violating all of the laws of dental economy. Besides, the most skillful cannot always be equal to the patient's needs. Nature will sometimes undermine the labors of the best. The pledge of a high and well merited reputation is always a sufficient guarantee to those who require your services. If such testimonials were necessary, those of the humblest carpenter, blacksmith or of any practical mechanic, would be far more competent than any of those just referred to. But you have no need of these, your well earned Degree of Doctor of Dental Surgery, embracing as it does, far more than it is possible for others justly to say, forever places you above the temptation or need of these questionable aids.

Never solicit patronage. You thereby degrade your profession to the level of a hawking trade, which goes begging for employment. Never be patronized, never speak of patrons, you should never have any. You should strive to be above patronage, by making your operations so superior that they will be above price. Who would ever think of *patronizing* a Liston, Velpeau or Mott! How is it possible to patronize a Maynard, Harris, Townsend or Dunning! As well might you speak of patronizing the physician who saved your life, or the surgeon who has restored your limbs, as to talk of patronizing the dento-artist who restores your perishing organs, models you into symmetrical form and saves you from the curse of deformity.

The public will sooner or later endorse the same estimate you place upon yourself, especially if it be a correct one, and you live up to it. Look well to this, and see that you neither deceive yourself or your friends. If you are equal to the high mark you have placed upon your ability, your services are invaluable, and no compensation can more than represent the commercial standard of value you place upon them; a large debt of gratitude, beyond price, remains behind, which, whenever appreciated—and it always will be sooner or later—is freely acknowledged; you earn for yourself an honorable name, and shed a glory and dignity upon your profession. But if you perform careless and and unskillful operations, and with hasty and heedless hand, abuse the confidence placed in your skill, only for the purpose of pecuniary gain, you are guilty of obtaining money under false pretences, and deserve to be indicted "according to the statute in such case made and provided." You degrade your high calling, do violence to the trust reposed in you, dishonor your manhood, and place yourself at the head of the army of imposters as the prince of quacks, for of all professional sins this is the most abominable, that of quackery in high places, the sin of apostacy from the true and acknowledged faith of our profession; a sin which with you can never have the redeeming feature of ignorance, nor of those dissenters who reject our faith while they openly avow another—they at least show their hand: but the intelligent apostate professedly adheres to good faith, while he outquacks the charlatan in practice.

Let me caution you never to use anesthetics in your practice, except under circumstances of extreme necessity, and then only in the presence of a third person; you owe this to your patients and your profession, as well as to yourselves. It becomes you to take warning from a fearful precedent recently established, which has characterized ours as an age of stupid degeneracy, and outraged justice, when the wild vagaries of dreams are acceptable evidence before juries, and the extravagant hallucinations of intoxication, qualify one for the witness stand.

Lengthy as this address has thus far been, I cannot bring it to a close, without urging upon you the importance of cultivating *self-reliance.* Depend upon yourselves, be the architects of your own fortunes: a man without self-reliance has no individuality, no character; he cannot even be said to be himself, if he ever had any unity of being, he has long since lost it; his nature is inverted, diffused and absorbed into others; it is distributed throughout the circle of his acquaintance, the largest share going to those who influence him the most; he is not of himself, but of others, and the aggregate of his personal identity is made up of scattered fractions. He has no centre within himself, and his mind is a chaos, without order or form; self-reliance would give him character and unity of being, and place within him a grand focus, towards which all his mental elements would turn and be subservient; it would be the electric energy which would resolve order out of his confusion. His opinions are his friends; his religion, his tastes and his politics are furnished beforehand, prescribed to his willing acceptance; his habits and morals are in charge of his fast friend, who, perchance, has his conscience, too, in keeping. "It soon becomes laborious for him to think, and he shrinks from its fatigue." Men who have no self-reliance are wavering and unsteady, they are dull and profitless, without sparkle or point. But men with great self-reliance and energy will season the world with it, and make it the rudder of their life, as Luther, Columbus, Napoleon. Energy, combined with self-reliance, are large ingredients in the characters of all great men.

Go out fearlessly into the field of your professional life, plunge the plough deep into the soil, and with a strong hand upheave the virgin earth, let the sun of science shine upon it, and experience, like the rains of heaven, mellow it—it shall quicken a glorious harvest for your reward. Have no such word as *fail!* what has been done can be done again—and *more*—and *you* can accomplish it. You do not know yourself what is within you; energy will develop it. Have faith in progress; it is one of the first principles of nature, and rules in eternity as here. Do not believe yourself if you have the coward thought of failure; believe in vital, electric *energy!* It has accomplished all that the world now is, and it underlies all that it ever will be. It hath leveled mountains, seamed the inland with mimic rivers, bound the continents with iron, ploughed the ocean "'gainst wind and tide," chained the lightning, and subdued all of the elements to our use. It hath done much, yet how much more may it not do for our art. The spirit of this indominable energy is in you all; each of you have at least a *one man power* of it in your natures; the same as did your neighbors of the past, as do your colaborers of the present, and as will those of the unborn future. It is there, if you did but know it, in all of its fullness; a *mighty energy*, such as nerved the arm and upheld the spirit of Cæsar, Cromwell, Gallileo, a Henry and a Washington. It is there, tempered by your spirit, but ready for action; rouse up these slumbering forces, and *let them out!* The world is the legitimate field of their action, and needs them all. It is your duty to let them out—they are hers. The world hath need of all these elements, and she prays for succor; she hath a wide field to be subdued, much rugged toil lies before her; forests to be cleared; cities to be built; she has suffering and woe to be soothed and stayed. The desert *must* blossom as the rose, and all of the nations of the earth must dwell together in one common brotherhood, before the great day of her delivery shall come, when she shall be crowned with the millenium! Enter the field and commence upon your part of the great mission.

In all of your relations and intercourse with life strive to enlarge your heart as well as your pocket; cultivate your humanity as well as your acres; with gathering harvests fill the swelling store-house of your mind, and build up in architectural beauty the aspiring temple of your soul! For the glittering dust shall be scattered by the winds, your acres shall be submerged or tilled by another, your earthly store-house be devoured by the elements or torn from you by misfortune, and your beauteous castle tumble to ruins. But your enlarged heart and christian humanity shall give you companionship with Christ and the chosen ones of his love; and, with a mind filled with virtue and all noble things, and soul expanded with immortal wisdom, you shall sit beside the throne of the Infinite, and enjoy his presence through a boundless eternity.

I would like to speak to you of success, what it is, and what it is not, but I must forbear, with only a few words.

I warn you that success is not abundant practice, nor fortune, nor eminence, unless you cultivate your heart and elevate your soul with it. Success is not thriving knavery, nor luck, that comes without labor or thought. Success is not what the world sees of the man, it is what the man *is* in himself. It depends upon what a man has done for his heart, for his immortal nature rather than for the earthly and perishing, which at best should only be aids to the development and growth of the soul "Every man can define to himself his own success; no other man can, for success is the great secret between man and his God," which eternity alone will reveal.

A dentist may be successful in his profession without contributing one iota to the general good of mankind; instead of plucking from the system the germinating seeds of disease, with superficial hand he may only have covered them over, leaving them to take deeper root, ultimately to spring forth with increased destructive energy.

A man may be eminently successful, according to the world's estimate, and his life be a grand and mournful failure.

And now let me speak of another element—the noblest element of success—indeed, there is no true success without it. I mean, the element of God's holy religion; "for what shall it avail a man if he gain the whole world and lose his own soul."

Look to heaven for support; you need it; we all need it. Oh, it is blasphemous arrogance to assume that we are equal to life *alone;* that we do not need God; that we have no sorrows for heaven to heal, no wounded hearts to be soothed; no need of God's pity, his grace, and his love; no pains or griefs or human woes for Christ to sympathize with and give us balm; no poor weakness that needs his strength—that we are equal to it all, and need not his sustaining grace, the blessing of his love, and the consolations of his spirit. Go daily and lay down your burden at the feet of the Lord, owning your littleness, your dependence and your need. And out of the abundance of your weakness shall you find strength; and because of the depths of your humility shall you be exalted. When the dark hours come, and the hand of affliction is laid upon you, you shall be comforted. In the exercise of your calling, and in all of your varied relations with the world, let your spirit daily commune with your God; and divine teaching and inspirations shall be given you, which, if heeded, will profit you practically, qualify you to meet all of the exigencies of life, and bless you forever.

